# Navigated TMS in the ICU: Introducing Motor Mapping to the Critical Care Setting

**DOI:** 10.3390/brainsci10121005

**Published:** 2020-12-18

**Authors:** Severin Schramm, Alexander F. Haddad, Lawrence Chyall, Sandro M. Krieg, Nico Sollmann, Phiroz E. Tarapore

**Affiliations:** 1 Department of Neurosurgery, Klinikum Rechts der Isar, Technische Universität München, Ismaninger Str. 22, 81675 Munich, Germany; Sandro.Krieg@tum.de; 2 Department of Neurosurgery, University of California San Francisco, 1001 Potrero Ave, San Francisco, CA 94110, USA; Alexander.Haddad@ucsf.edu (A.F.H.); Lawrence.Chyall@sfdph.org (L.C.); Phiroz.Tarapore@ucsf.edu (P.E.T.); 3 TUM-Neuroimaging Center, Klinikum Rechts der Isar, Technische Universität München, 81675 Munich, Germany; Nico.Sollmann@tum.de; 4 Department of Diagnostic and Interventional Neuroradiology, Klinikum Rechts der Isar, Technische Universität München, Ismaninger Str. 22, 81675 Munich, Germany

**Keywords:** nTMS, brain stimulation, intensive care, motor mapping, ICU, neurocritical care, neuromonitoring, functional mapping, motor evoked potentials

## Abstract

Navigated transcranial magnetic stimulation (nTMS) is a modality for noninvasive cortical mapping. Specifically, nTMS motor mapping is an objective measure of motor function, offering quantitative diagnostic information regardless of subject cooperation or consciousness. Thus far, it has mostly been restricted to the outpatient setting. This study evaluates the feasibility of nTMS motor mapping in the intensive care unit (ICU) setting and solves the challenges encountered in this special environment. We compared neuronavigation based on computed tomography (CT) and magnetic resonance imaging (MRI). We performed motor mappings in neurocritical patients under varying conditions (e.g., sedation or hemicraniectomy). Furthermore, we identified ways of minimizing electromyography (EMG) noise in the interference-rich ICU environment. Motor mapping was performed in 21 patients (six females, median age: 69 years). In 18 patients, motor evoked potentials (MEPs) were obtained. In three patients, MEPs could not be evoked. No adverse reactions occurred. We found CT to offer a comparable neuronavigation to MRI (CT maximum e-field 52 ± 14 V/m vs. MRI maximum e-field 52 ± 11 V/m; *p* = 0.6574). We detailed EMG noise reduction methods and found that propofol sedation of up to 80 mcg/kg/h did not inhibit MEPs. Yet, nTMS equipment interfered with exposed pulse oximetry. nTMS motor mapping application and use was illustrated in three clinical cases. In conclusion, we present an approach for the safe and reliable use of nTMS motor mapping in the ICU setting and outline possible benefits. Our findings support further studies regarding the clinical value of nTMS in critical care settings.

## 1. Introduction

Navigated transcranial magnetic stimulation (nTMS) is an established technique for non-invasive brain stimulation [1,2]. At present, the technique is commonly applied in pre-operative mappings of patients undergoing neurosurgical procedures. In this study, we investigate whether nTMS may also play a role in the diagnosis and management of acute neurological injury in the inpatient setting. Because it is image-guided (and therefore highly accurate), it can provide detailed information about the level of intactness in specific neuromuscular pathways (useful in spinal cord injury). Unlike transcranial electrical motor-evoked potentials, it is non-invasive and painless, and can therefore be applied at bedside without anesthesia. Furthermore, it does not require patient participation, and may thus be used in patients who are sedated, or who are awake but unable to participate in detailed neurological examination. For these reasons, nTMS, if it can be successfully implemented in the neurocritical care setting, would offer a valuable new method for quantifiable neurological examination in patients with suspected or known neurological compromise.

TMS involves a strong magnetic field pulse that penetrates the skull and causes depolarization of underlying neuronal tissue via the principle of electromagnetic induction. When using a figure-of-eight coil it is highly focal and entirely noninvasive [2,3]. By combining the capacity for focal cortical stimulation with image-based, frameless stereotactic neuronavigation, nTMS allows for highly accurate, navigated interrogation of cortical function [1,4]. The broad applicability of this basic principle has created applications for nTMS in both the basic science and clinical realms. The technique has been utilized across neuroscience disciplines such as psychiatry, in physical medicine and rehabilitation, and neurosurgery [5,6,7].

As mentioned earlier, a common application of nTMS is the mapping of the primary motor cortical system (MCS). Here, stimulation of the cortical motor area leads to peripheral muscle activation (motor evoked potentials [MEPs]), which is recorded via electromyography (EMG) [1]. This process allows for attribution of a specific muscle response (i.e., abductor pollicis brevis or tibialis anterior muscles) to its respective cortical location, thereby generating an individualized functional map of the MCS [8,9]. Herein, the navigational aspect adds critical capabilities to the underlying TMS technology. Compared to non-navigated TMS of the motor cortex, where the stimulation site is usually determined in relation to anatomical landmarks [10], nTMS has been shown to be superior in evoking replicable and high-amplitude MEPs [11]. Importantly, nTMS allows optimal orientation of the electric field in relation to individual gyral and skull anatomy, a critical aspect in ensuring ideal MEP generation [12]. Furthermore, for any form of longitudinal monitoring, precise revisiting of previously identified sites is vital and, therefore, necessitates accurate navigation. The accuracy of nTMS motor mapping is superior to other noninvasive techniques such as functional magnetic resonance imaging (fMRI) and magnetoencephalography (MEG), and is highly comparable in accuracy to intraoperative direct cortical stimulation (DCS), the current gold-standard method for functional mapping [1,13].

In the clinical context, mapping of the MCS is often performed in preparation for brain tumor surgery, allowing for better presurgical planning by identifying eloquent motor regions and their relationship to the lesion [5,9,14,15]. While increasingly common in the outpatient setting, nTMS motor mapping has thus far remained underutilized in the acute inpatient setting. The use of other neurophysiological monitoring modalities in the ICU, most notably electroencephalography (EEG), has opened up a range of diagnostic innovations in recent years [16,17,18,19,20]. Similarly, nTMS could be a valuable tool for the inpatient setting. It can provide a highly accurate, low-cost, and relatively quick assessment of the functionality of the MCS without dedicated subject participation. It would thus be the ideal modality for evaluation of the corticospinal integrity and excitability in patients with altered mental status or coma. Alternatively, because it is painless, nTMS may also be used to assess the MCS in patients who are awake but unable or unwilling to participate in neurological examination.

However, to date, only few studies have addressed the application of nTMS motor diagnostics in the ICU context. While TMS/EEG combination setups have been used in exploring disorders of consciousness [20], nTMS motor mappings in the intensive care framework are at this time not reported in the literature. The reason for the current lack of exploration is likely the plethora of challenges faced in translating established protocols and workflows to the unique setting and patient population found in the ICU (Table 1). This study seeks to explore the feasibility of nTMS motor mappings in the intensive care framework, based on established protocol and safety guidelines [8,21]. By identifying the primary challenges, working through solutions, and sharing our results, we hope to encourage and enable further studies into the value of nTMS motor mapping in the neurocritical care setting.

## 2. Materials and Methods

### 2.1. Study Setup and Patient Inclusion

This study was approved by the UCSF Institutional Review Board (16-20684, 1/9/2020). All research was conducted according to the Declaration of Helsinki. Informed consent was acquired from conscious patients or next of kin when known.

We conducted nTMS motor mappings on patients treated in the neurological ICU of Zuckerberg San Francisco General Hospital. Patients are referred to this unit when neurological damage necessitated continuous monitoring of vital parameters, close interval neurochecks, mechanical ventilation, vasoactive infusions, and other typical ICU indications. Available and commonly applied monitoring includes blood pressure (both invasive and noninvasive), pulse oximetry, electrocardiography (ECG), intracranial pressure (ICP), EEG, and interval neurochecks [22].

Inclusion criteria were defined as treatment in the ICU, confirmed presence of brain or spinal lesion and suspicion or presence of a motor deficit due to neurological damage. Exclusion criteria were denial of consent by patient or next of kin, uncontrolled epilepsy, presence of fixed nTMS-interacting devices close to or within the skull (e.g., cochlear implant, deep brain stimulation), acute isolation due to infectious disease, acute instability of vital parameters, and end-of-life care. The procedures were explained to the patient (if conscious) and/or next of kin (if known).

The nTMS system was deployed into the patient’s room on the ICU, and motor mapping was performed. After the examination, clinical factors such as ICP and sedation were documented if applicable. Afterwards, the data were analyzed by experienced nTMS users (SS, PT) and a report on the procedure and results was written for internal use.

### 2.2. Image Acquisition and Processing

All CT imaging in this study was acquired with a Somatom 64-slice CT scanner (Siemens AG, Erlangen, Germany), all MRI imaging with a Magnetom Skyra 3T MRI scanner (Siemens AG, Erlangen, Germany). In order to compare the navigational accuracy of CT and MRI, we exported image stacks of both CT and MRI from 4 patients that had received both types of scans. For CT, we exported 4 scans of 2 mm slice thickness (3 sagittal, 1 axial). For MRI, we exported 4 T1-weighted scans with slice thickness ranging from 0.75 mm to 5 mm (3 sagittal, 1 axial).

**Figure 1:**
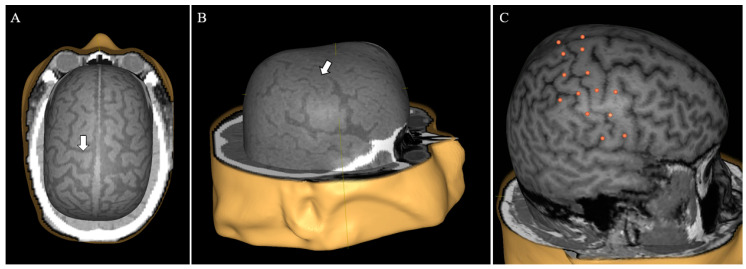
Head model based on computed tomography (CT). By using appropriate preprocessing, CT can serve as a viable basis for head model reconstruction in the nTMS system (**A**,**B**). Important anatomical structures such as the precentral gyrus are easy to identify (**A**,**B**, white arrows). For image testing, 12 targets were placed around the central sulcus on each hemisphere with two additional targets at the midline vertex (26 targets total per brain, **C**).

To make CT scans viable for use in the nTMS system, we performed a series of preprocessing steps (removal of non-patient structures such as e.g., headrest, proper windowing, intensity rescaling) using the Aliza Medical Imaging and DICOM Viewer (Aliza 1.98.12, Copyright 2014–2020 Aliza Medical Imaging, Bonn, Germany), the details of which are available as Supplementary Material (Document S1). The exported DICOM files were loaded into the nTMS system (Nexstim eXimia NBS system, version 4.0; Nexstim Plc., Helsinki, Finland) and used to create head models (Figure 1A,B).

### 2.3. CT/MRI Comparison

In order to validate the use of CT imaging compared to the standard MRI imaging, we compared the e-field calculated by the system in both modalities in four sets of imaging data. For each head model, 26 stimulation targets were put on the cortical surface, distributed around the central sulcus (Figure 1C). The virtual model was co-registered to a dummy head in 3 cases and to the actual patient in 1 case. Each target was subsequently stimulated with an intensity of 30% of maximum stimulator output and the calculated local maximum e-field was recorded. This specific stimulation intensity was selected since 30% of maximum stimulator output is a common benchmark for motor thresholds in neurosurgical motor mappings.

### 2.4. Motor Mapping Protocol

For patient motor mappings, we followed the existing guidelines [8]. We employed an e-field-navigated TMS system (Nexstim eXimia NBS system, version 5.0; Nexstim Plc., Helsinki, Finland). The system provides an integration of all necessary subcomponents (navigation, figure-of-eight stimulation coil, EMG). For EMG recordings, surface electrodes were used (Neuroline 700; Ambu, Ballerup, Denmark). The nTMS system was positioned next to the patient’s bed. The patient’s skin at electrode placement spots was cleaned with alcohol swabs. EMG electrodes were applied to all muscles of interest, chosen according to the lesion location and predicted or known motor deficits.

EMG noise optimization was performed by finding optimal electrode placement spots, avoiding crossings of cables and eliminating all non-essential sources of electromagnetic noise [23]. Recorded muscles for the upper extremity were deltoid muscle, biceps brachii muscle, flexor carpi radialis muscle, and either abductor pollicis brevis muscle or adductor digiti minimi muscle, depending on accessibility. For lower extremities, recorded muscles were quadriceps femoris muscle, tibialis anterior muscle, and gastrocnemius muscle. MEPs were considered valid according to amplitude (above 50 µV), latency (10–35 ms for upper extremity musculature, 30–60 ms for lower limb musculature) [24], and plausible morphology.

Prepared imaging was loaded into the system and a head model was created. After successful co-registration, the head model was peeled to an individual depth allowing for visual identification of the precentral gyrus. During the entire mapping, the coil was positioned tangentially to local skull surface, aided by the neuronavigation system [3]. E-field orientation was kept at 90 degrees to local gyrus orientation [1]. A rough mapping was performed to give primary information on muscle representations of the upper extremity and possible motor hotspots. After identification of a given motor hotspot, said point was used for resting motor threshold (rMT) determination, defined as the stimulation intensity that results in a valid MEP in 50% of cases [8]. rMT determination was performed via a system-integrated procedure based on the maximum likelihood algorithm [25,26].

After determination of the rMT, the full extent of the MCS was mapped with 105% rMT. Then, lower extremity musculature was mapped with an intensity starting at 130% rMT. The distance between individual stimuli was roughly 5 mm. If no MEPs could be elicited, the stimulation intensity was raised stepwise until either MEPs were present, the patient reported discomfort, or no further raise was possible. For patients with prior hemicraniectomy (consisting of the temporary removal of a large fronto-temporo-parietal skull flap to allow for decompression of brain tissue) intensity was capped at 75% of maximum stimulator output due to safety considerations. In post-hoc analysis of motor mappings, the amplitude of EMG noise after signal optimization was measured and noted down for each mapped muscle for later descriptive statistical analysis.

The primary maxim during motor mapping was to avoid disrupting the nursing routine or clinical necessities. Extraordinary effort was made to explain the testing procedure to the bedside nurses and to ensure that testing would not disrupt their patient care responsibilities. Emphasis was made on the importance of open communication between the nursing team and the nTMS team. During testing, no active monitoring was removed from the patient and care was taken to minimize patient mobilization. If any other procedures had to be performed on the patient (e.g., neurological examination, suction, repositioning, and toileting), the examination was paused or aborted. After the examination, all nTMS equipment was removed from the patient, the room was returned to its original layout, and the system was cleaned and brought back to its designated storage place.

### 2.5. Statistics

Statistical analysis was performed in GraphPad Prism (version 8.0.0, GraphPad Software, San Diego, CA, USA). Due to the lack of prior investigations in the literature, we were hesitant to assume any given data distribution. Therefore, we tested for statistically significant differences between CT- and MRI-based e-field maxima using the non-parametric Wilcoxon matched-pairs signed rank test. The level of statistical significance was defined as *p* < 0.05.

## 3. Results

### 3.1. Patients

We performed motor mappings by nTMS in a group of 21 patients (6 females) with a range of diagnoses and etiologies (Table 2). Median age was 69 years (age range: 17–89 years). Seven patients were mapped under sedation via propofol (median dosage: 30 mcg/kg/min, dosage range: 15–80 mcg/kg/min). Nine patients underwent motor mapping of a hemisphere ipsilateral to hemicraniectomy. Five patients had ICP monitoring in place (two via drainage, three via cranial bolt). Average ICP in these cases was 15 ± 4 mmHg (median ICP: 17 mmHG, range ICP: 9–18 mmHg).

### 3.2. Imaging Testing Results

The average difference of CT to MRI e-fields was −0.4 V/m ± 7.5 V/m (median difference: 0 V/m), and the maximum difference in e-fields between corresponding points was 18 V/m. In our statistical analysis of recorded e-field values, we found no statistically significant difference (mean CT e-field: 52 ± 14 V/m vs. mean MRI e-field: 52 ± 11 V/m; *p* = 0.6574).

### 3.3. Mapping Results

MEPs were successfully evoked in 18 patients. Average rMT (in percent of maximum stimulator output) was 63 ± 20% (median rMT: 68%, rMT range: 28–91%) for left hemisphere and 62 ± 22% (median rMT: 63%, rMT range: 31–100%) for right hemisphere. Within the sedated group of patients, MEPs were successfully evoked in 5 out of 7 patients, including the two cases with the highest observed sedation levels of 80 mcg/kg/min and 70 mcg/kg/min propofol, respectively. Excluding image preparation, time required per mapped hemisphere was approximately 90 min.

There were no adverse effects during or immediately after motor mapping. Conscious patients did not report the stimulation to be painful or uncomfortable. We observed no changes in continuously monitored autonomic parameters (O_2_ saturation, blood pressure, heart frequency, ICP) attributable to stimulation. nTMS motor mapping did not prevent any other necessary procedure (e.g., neurochecks, application of medication, and suction) from being carried out. If necessary, the mapping was paused until the end of the procedure.

Intracranial access devices (e.g., subdural drainage and cranial bolt) interfered with motor mappings due to physical limitations imposed on coil placement. Cranial bolts specifically blocked access to sections of the respective hemisphere by taking up space. Motor mapping in these patients was, therefore, restricted to the hemisphere contralateral to the cranial bolt. ICP monitoring via drainages complicated but did not prohibit mappings of the ipsilateral cortex.

**Figure 2:**
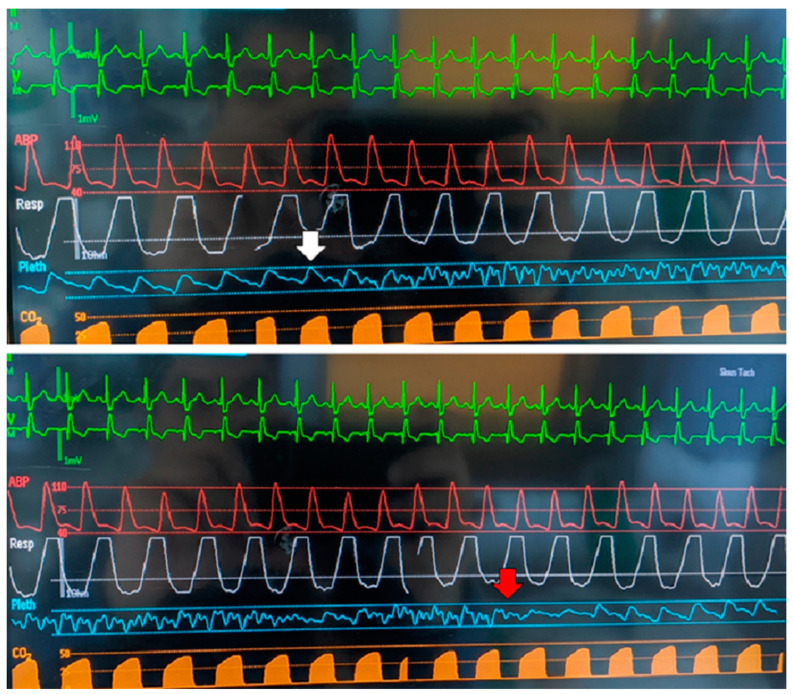
Interference of navigation equipment with pulse oximetry. This figure demonstrates the effect that the navigated transcranial magnetic stimulation (nTMS) navigational system imposes on pulse oximetry. The underlying mechanism is likely the infrared-spectrum based navigational camera creating interference with light used in pulse oximetry. The white arrow indicates the moment at which the camera was positioned for nTMS navigation. The red arrow indicates the moment at which a layer of textiles was put over the pulse oximetry probe.

Additionally, we were able to demonstrate that nTMS navigation equipment interferes with pulse oximetry (Figure 2). This problem was solved by putting textiles over the probe, thereby blocking the interfering signal.

### 3.4. EMG Noise Results

**Figure 3:**
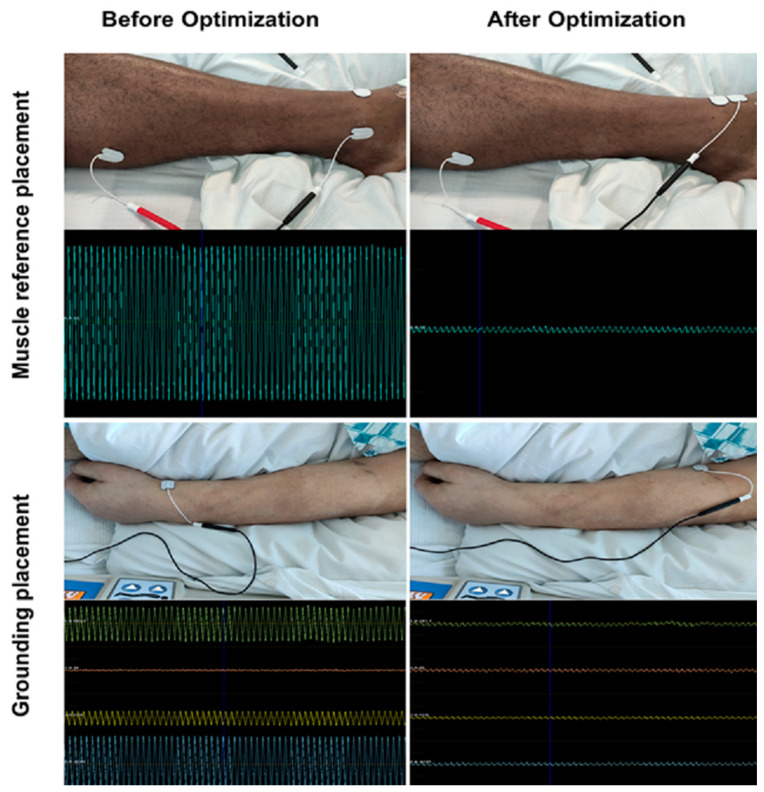
Electromyography (EMG) noise optimization. This figure illustrates the effect of electrode placement on noise level. All placement spots are common for neutral electrode placement, yet significant differences can be observed. At bedside, optimal positioning often requires testing of different spots until an adequate noise level is achieved. EMG scales are equal within the same row.

**Figure 4:**
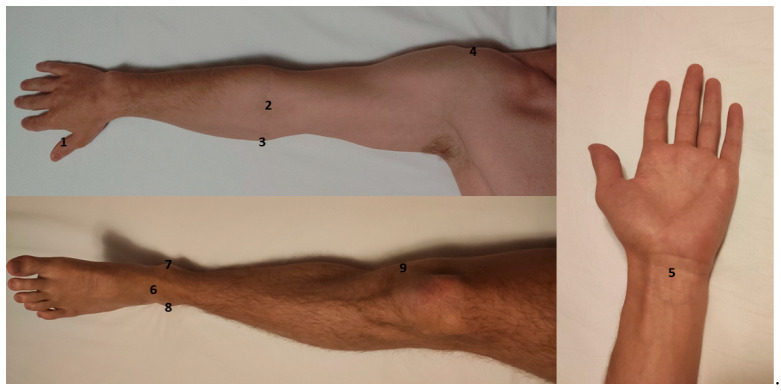
Placement spots for neutral electrodes. This figure illustrates anatomic landmarks that often emerged as viable for neutral electrode placement. 1: medial and lateral side of thumb, interphalangeal joint; 2: tendon of biceps brachii muscle; 3: medial epicondyle of humerus; 4: acromion; 5: tendons of hand flexors; 6: tendons of foot extensors; 7: medial malleolus of tibia; 8: lateral malleolus of fibula; 9: medial epicondyle of femur.

High amounts of noise were very common for patients in the ICU. With careful selection of electrode placement, however, it was usually possible to optimize noise sufficiently to allow for motor mapping (Figure 3). Average noise amplitude after optimization was 64 ± 58 µV (median noise amplitude: 43.5 µV). Trial-and-error approaches were often required for successful optimization, which entailed up to 10 different electrode placement trials per muscle. For each muscle group, particular anatomic locations for electrode placement (muscle reference electrode and grounding electrode) emerged. Despite identifying these sites as having a higher probability for noise mitigation, optimization via trial and error was nevertheless required in most cases (Figure 4). In many cases, noise optimization was the most time-consuming part of the procedure.

### 3.5. Illustrative Patient Cases

#### 3.5.1. Sixty-One-Year-Old Male with Subarachnoid/Subdural Hemorrhage

A 61-year-old male presenting with left-sided subdural and subarachnoid hemorrhage after a fall was transferred to ICU. For clinical management, left-sided hemicraniectomy and sedation were required, the latter of which kept the patient in a comatose state. Due to asymmetric reaction to painful stimuli (left limbs moving against gravity, no movement on right side), motor mapping by nTMS was performed on the left hemisphere to test MCS function (right hemisphere was blocked due to cranial bolt). In contrast to the clinical examination, we were able to demonstrate intact MEPs in the adductor digiti minimi muscle (rMT: 36% of maximum stimulator output). Furthermore, by increasing mapping intensity to 125% of rMT, we observed additional MEPs in biceps brachii and flexor carpi radialis muscles, indicating that corticospinal tract innervation of those muscle groups was intact. As a result of this map, additional workup (CT angiography, EEG, MRI) of the patient’s asymmetric examination was deferred. A follow-up motor mapping performed three days later showed a decrease in rMT to 28% of maximum stimulator output, indicating normalization of MCS threshold. Additionally, MEPs for tibialis anterior and gastrocnemius muscles were now detectable. Volitional movement started to return in the clinical examination six days after the initial mapping with slight withdrawals from painful stimuli. At discharge, 14 days after initial motor mapping, the patient displayed symptoms of hemineglect, but was able to move all extremities spontaneously.

#### 3.5.2. Sixty-Seven-Year-Old Male with C1 Ring Fracture

A 67-year-old male patient presenting with comminuted/displaced C1 ring fracture after assault was treated on the ICU, presenting without any initial neurological symptoms. The patient underwent stabilization surgery, after which he developed aspiration pneumonia with acute respiratory distress syndrome (ARDS), necessitating prolonged sedation and ventilation under medically induced paralysis. Seventeen days after admission, the patient’s ARDS improved and the paralysis was stopped. After the paralytics had worn off (as determined by intact train-of-four), the patient would open their eyes and track movement but remained unresponsive to central or peripheral stimulation. Motor mapping by nTMS was performed for both hemispheres. Despite the lack of movement in the clinical examination and a concurrent propofol infusion (10 mcg/kg/min), we were able to demonstrate MEPs in at least one muscle for every limb, albeit with very high rMT (81% and 99% of maximum stimulator output for left and right hemisphere, respectively). These findings effectively ruled out an occult spinal cord injury, and as a result, further workup with MRI and EEG was deferred. The next day, in a follow-up study with increased propofol dosage (40 mcg/kg/min), MEPs disappeared from both upper limbs and the left lower limb. The right lower limb still demonstrated MEPs but required a higher stimulation intensity of 100% versus 90% on the day before. These changes were thought to be a result of the increased propofol dose. In the subsequent days, the patient level of consciousness improved but he remained unable to follow commands or move volitionally, which was attributed to myoneuropathy of critical illness. Shortly thereafter, the patient developed a bowel perforation, and died of septic shock.

#### 3.5.3. Seventy-Seven-Year-Old Male with Subdural Hematoma

A 77-year-old male was referred to the ICU with left holohemispheric subdural hematoma and midpontine hemorrhage after a fall. The patient remained non-verbal during his entire treatment. At admission, the patient did not follow commands and displayed hemiparesis on his right side but showed flexion to painful stimulation with his left side. The patient’s family declined immediate surgical intervention, instead opting to wait for potential improvement over time. Four days after admission, the patient started following commands from his wife, but continued to only move his left side. Eight days after admission, the neurological state of the patient deteriorated, and he demonstrated left-sided decerebrate posturing. CT imaging demonstrated an increase in the size of the subdural hematoma and subfalcine as well as uncal herniation. After consulting with the patient’s family, hemicraniectomy and evacuation of the hematoma were performed over the left hemisphere. Two days after the procedure, nTMS motor mapping was performed due to continued absence of movement on the right side. Contrary to the clinical presentation, MEPs were present bilaterally for both upper and lower extremities. Notably, the rMT on the left hemisphere was higher (42% of maximum stimulator output) than the rMT on the right hemisphere (34% of maximum stimulator output), despite the left sided hemicraniectomy. The results were therefore interpreted as pointing towards a damaged but still functional MCS, with hemineglect as a potential explanation for the present symptoms. Physical therapy, therefore, emphasized maintaining right-sided range of motion and targeted therapy to address right hemi-neglect. During this time, patient began to regain motor function on his right side. A follow-up mapping took place 8 days after the inital map. At this point, the patient had received cranioplasty and demonstrated an rMT of 37% of maximum stimulator output for the right hemisphere and 62% of maximum stimulator output on the left hemisphere. The patient’s rehabilitation continued, his neglect improved, and he eventually demonstrated 4/5 muscle strength throughout his right side. Upon his discharge 26 days after admission, he was intermittently following commands, moving all his extremities purposefully, and was able to continue his physical and cognitive therapy in an acute rehabiliation institution.

## 4. Discussion

We performed nTMS motor mappings in 21 patients being treated in the neurological ICU for a variety of neurological injuries. All mappings were successfully and safely completed. In so doing, we identified common problems that occur in the ICU setting and, where possible, we arrived at solutions for these problems. This feasibility study will enable future studies to focus on establishing the clinical value nTMS-based motor mappings in the ICU and, more broadly, in the inpatient setting.

### 4.1. Safety

As with any new clinical test, particularly when dealing with the critically ill, patient safety is of utmost importance. nTMS in general is widely considered a safe modality, especially when single-pulse protocols are used [5,21]. The most serious adverse effect associated with TMS is the occurrence of epileptic seizures. These, however, are exceedingly rare, limited to isolated case reports often in individuals with prior history of epilepsy [27,28]. While we did exclude from our study any patient with uncontrolled epilepsy, none of our patients showed any adverse effects during or immediately after single-pulse nTMS motor mapping. No acute worsening of clinical state was attributable to motor mapping. This was also true for the nine patients who underwent mapping following prior hemicraniectomy, in whom the magnetic field incident upon the cortex is likely stronger than for patients with an intact skull. It should be noted again, however, that we capped the stimulation intensity for hemicraniectomy hemispheres to 75% of maximum stimulator output for precautionary reasons. Although these data are gathered from a limited number of cases, it is highly encouraging and indicates that nTMS is likely safe enough to be performed in patients with severe acute neurological damage and altered cerebral anatomy.

### 4.2. Neuronavigation Based on CT

A prerequisite for nTMS motor mapping is preexisting cranial imaging used for neuronavigation. In the conventional workflow, MRI offers unparalleled anatomical imaging of brain tissue with a high soft tissue contrast, and it is, therefore, the gold standard for navigational imaging [8]. MRI, however, is oftentimes not a viable option for ICU patients due to practical considerations or contraindications for scanning. Specifically, critically ill patients are often unable to tolerate lengthy MRI studies. Furthermore, intracranial monitoring devices such as cranial bolts and indwelling electrodes can be incompatible with the scanning environment [29]. Although it offers less detailed imaging of intracranial anatomy than MRI, CT is by far the more prevalent modality for patients in the ICU due to its rapid acquisition time and compatibility with clinical equipment [29,30]. Therefore, to enable widespread use of nTMS motor mapping in the ICU, we realized that CT must be adapted and validated as the basis for neuronavigation. Using CT for this purpose has previously only been described in a singular recent case which did not address possible navigation discrepancies between CT and MRI [31]. By preprocessing CT scans with a slice thickness of 2 mm, we were able to generate usable head models suited for patient co-registration (Figure 1). Comparing the calculated maximum e-field at corresponding points in CT head models to that in MRI head models, we found no significant difference in calculated e-field values. While the maximum difference between two points was 18 V/m, we believe that this discrepancy was likely an artifact due to the inherent difficulty of co-registering a given head model to a physical dummy with different measurements. Our results indicate that both CT and MRI can yield comparable neuronavigation in the employed nTMS system. Following our process enables targeted, replicable stimulation of given brain loci in patients lacking MRI data. This application of CT-based neuronavigation not only makes motor mapping in the ICU an option, but also extends nTMS usage to any patient unable to undergo MRI (because of retained metal fragments, implanted devices, etc.).

### 4.3. Compatibility with ICU Workflows

In our cases, translation of the outpatient workflow into the ICU setting did, for the most part, not pose significant problems. Nursing routines or monitoring were not impeded. In this regard, the flexibility of nTMS motor mappings is a relevant asset, as the examination takes place at the bedside and can be paused at any moment. Pulse oximetry, while being influenced by navigational equipment (Figure 2), can easily be maintained by physically covering the oximetry probe. The only major limitation was imposed by immovable devices connected to the patient’s skull (i.e., cranial bolts placed for anchoring invasive ICP monitoring), as they tended to physically obstruct free movement of the coil. Overall, our experience demonstrated that nTMS motor mapping is compatible with the monitoring setup and clinical routine in ICU cases.

### 4.4. Optimization of EMG

**Figure 5:**
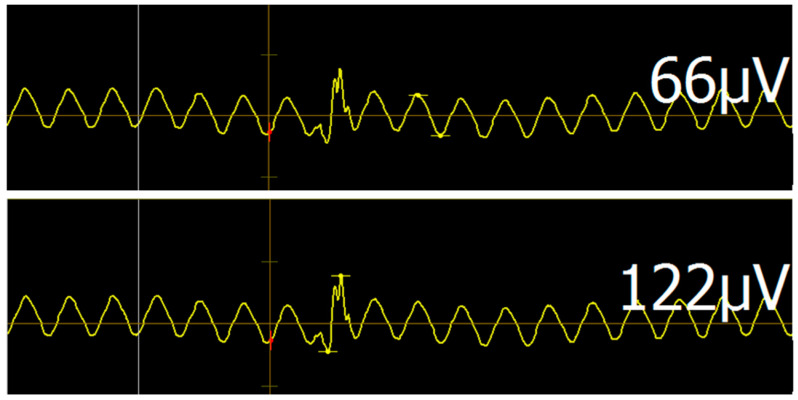
Motor evoked potential (MEP) disrupts 60 Hz noise. This figure shows regular 60 Hz noise disrupted by MEP occurrence. Measurements on the right correspond to amplitude between yellow measurement bars. One heuristic for detecting valid MEPs amongst regular noise is to subtract the noise amplitude from the potential MEP. If the amplitude is still >50 µV, the MEP may be considered valid (pictured). Additionally, placement of multiple stimuli at a given location can aid when MEPs are questionable. Note the two individual peaks within the MEP formed by the underlying noise reaching its minimum.

For reliable and accurate motor maps, clear EMG readings are necessary to detect even MEPs with small amplitudes. Any active electrical device in proximity to the recording site (e.g., ventilators, perfusors, and monitoring) is a potential source of noise [23]. It is, therefore, unsurprising that noise levels in the ICU setting are far higher than in the normal outpatient setting. Usually, electronic noise presents as a uniform waveform with frequency of about 60 Hz (corresponding to the 60 Hz alternating current of standard US power outlets). Elimination of noise by deactivation of electrical devices is for the most part impossible since their continued functioning is essential for patient care. While this noise generally allows for MEP detection via disruption of the regular noise pattern (Figure 5), maximum signal-to-noise ratio is required to ensure validity and comparability of individual mappings. To minimize noise, general guiding principles of EMG should be followed, such as avoiding contacts between individual cables, cleaning the skin prior to electrode placement, and ensuring full surface contact of electrodes with skin.

The most important factor in our study, however, seemed to be the location of electrode placement, particularly regarding reference and grounding electrodes (Figure 3). Established anatomical landmarks that work in the outpatient setting may not work in the intensive care case. Anatomical positions often proved to be successful neutral electrode targets in this study (Figure 4). In our cases, we were able to achieve an average noise amplitude of 64 ± 58 µV. This distribution is slightly skewed by a few cases with persistent high-amplitude noise levels, which is reflected in the median level of 43.5 µV. Analyzing data at this level of noise is possible, since any valid MEP (threshold of 50 µV) will peak through the background noise. In higher noise levels, however, analysis gradually loses sensitivity and it becomes difficult to detect low-amplitude MEPs. Although this noise lowers the resolution of motor mappings, useful information regarding binary questions of motor system integrity (such as “is any activation present?”) can still be obtained. One heuristic for detecting valid MEPs amongst regular noise is to subtract noise amplitude from the potential MEP. If the amplitude is still >50 µV, the MEP may be considered valid (Figure 5). Placement of multiple stimuli at a given location to verify replicability is also advisable in cases of questionable MEPs.

### 4.5. Pharmacological and Physiological Confounders

Patients in the ICU are subject to a variety of factors that most likely affect MEP elicitability, such as elevated ICP, hemicraniectomy, and propofol sedation. Propofol has previously been shown to decrease MEP amplitude, yet without impacting MEP latency [32,33,34]. One study compared the influence of different anesthetic agents on MEPs in animals and humans. The results indicated that even in high concentrations, propofol was unable to fully inhibit MEPs elicited by higher-than-threshold stimulation [34]. Additionally, it has been demonstrated that intravenous agents such as propofol impact MEPs less than volatile anesthetics [35]. The highest sedation levels in our study were propofol infusion of 80 mcg/kg/min and 70 mcg/kg/min. These doses did not prevent elicitation of MEPs in either case. While other patients may require higher doses, the current findings fit to the aforementioned literature, which indicates that MEP monitoring is still possible under propofol sedation. Based on the literature and our experience, we would recommend that patients under sedation showing no MEPs at 105% rMT should be tested with progressively higher stimulation intensities. While not tested in our approach, the literature indicates that TMS with repetitive pulse patterns might also be an option to elicit reliable MEPs under propofol sedation [34,36]. The dose-dependent input/output relationship between stimulus intensity and MEP amplitude might be exploited for individualized functional anesthesia monitoring. For nTMS applications in the ICU that are not specifically related to the MCS, it should be kept in mind that higher-order neuronal function such as network complexity are influenced by propofol as well [37]. Of note, opioids seem to only possess a small effect on MEPs, and we found no dampening of MEP due to opioids in our series [35].

Furthermore, ICP is another factor that could plausibly influence MEPs. Persistently elevated ICP is a known predictor of poor neurological outcome, as it lowers cerebral perfusion pressure resulting in cerebral hypoxia [38,39]. These effects are primarily observed at sustained ICPs of 25 mmHg and higher [38]. All of our cases were performed with an ICP lower than 20 mmHg, as patients are treated aggressively to maintain an ICP below this value. Therefore, while we can state that MEPs were still elicitable in the highest observed ICP of 18 mmHg, we can make no claims as to the influence of ICPs in higher ranges. Hemicraniectomy is a procedure often utilized to combat elevated ICP [40,41], and nine of our patients had undergone this procedure. While our main concern in mapping patients after hemicraniectomy was patient safety, it is worth reporting that MEPs could still be elicited in this abnormal anatomical state.

### 4.6. Implications and Applications

We believe that our reported findings serve as valid evidence for the general feasibility of nTMS motor mappings in the intensive care setting. In our series, we were able to map successfully 18 out of 21 patients, yielding reproducible MEPs, and resulting in no complications. These findings demonstrate that, for the critically ill patient with neurological injury, nTMS can deliver quantitative analysis of MCS functionality in a safe and reliable manner. nTMS motor mapping allows for objective investigations into the integrity of the motor system along its entire path, from upper motor neurons to lower motor neurons, and thereafter to the neuromuscular junction. Importantly, it does not rely on patient consciousness or cooperation. Furthermore, rMT specifically has been described to reflect neuronal excitability, which may be a metric of interest in a variety of acute neurological problems [42]. One example is traumatic brain injury, which has been linked to rises in rMT [43].

The potential applications and benefits of nTMS motor mapping on the ICU are numerous. Examinations can be performed on conscious and unconscious patients alike and yield detailed information about the function of individual corticospinal tracts. The procedure is painless, noninvasive and does not require (yet is possible during) sedation. Additionally, any trained member of the clinical team can perform a mapping without the need for additional personnel.

Compared to other, more established neurophysiological modalities such as EEG or somatosensory evoked potentials, nTMS offers the unique capability of linking effects to a specific, repeatedly targetable cortical site [16]. This enables detailed tracking of functional MCS changes over time, which is not present with non-navigated TMS (due to targeting inaccuracy) or EEG methods. Earlier diagnosis of specific motor deficits and prediction of motor recovery may be possible, since corticospinal integrity can be objectively demonstrated for any EMG-suited target muscle. For example, MEP recovery has recently been shown to predict neurological outcome in endovascular thrombectomy [44]. Muscular recruitment curves could serve as the basis of a standardized measure of cortical excitability. In a similar vein, individualized functional drug monitoring specifically for antiepileptic drugs or anesthesia seems plausible [33,45,46,47]. Protocols for specific types of monitoring could potentially be used for recognition and monitoring of transient complications, such as vasospasm in subarachnoid hemorrhage.

Of note, in all three clinical cases introduced above, nTMS motor mapping yielded information on the acute state of the MCS that was not visible in the clinical examination by demonstrating functional corticospinal motor connection despite lack of movement.

### 4.7. Limitations

While our findings confirm the general feasibility of nTMS motor mappings in the ICU setting and demonstrate some use cases, there are several limitations that need to be addressed. First, this study includes only 21 unique patients, which may be considered a small sample size. However, as a pilot feasibility study, we are confident that this sample is adequate to provide general guiding principles that may be built upon in future studies. Second, our imaging testing was limited both by number of cases and by the margin of error in registering a head model to a dummy head of different size. We believe, however, that with 104 total targets per modality, we would have been able to discover any meaningful systematic difference in e-field calculation if it had existed. A separate aspect of interest would be to investigate the comparability of CT and MRI not only based on calculated e-field, but also on physical coil position, which would require some form of system-separate coordinate measurement. For a more extensive comparison, a large set of cases with CT and MRI datasets acquired within a small timeframe would be necessary. Since CT is inherently connected to radiation exposure, a study such as this could hardly be done with healthy volunteers. Even with patients, however, indications for both imaging modalities in short intervals remain rare, which unfortunately limits the pool of usable data. In conclusion, while the presented evidence may be lacking in robustness, it serves as a basis for more extensive systematic testing and, more importantly, demonstrates that repeatable targeting of specific cortical stimulation sites is possible with CT imaging. Third, this study took place in a single ICU at a single institution. Other centers may encounter other challenges based on respective local workflow or electrical noise environment. Fourth, in terms of clinical factors influencing MEPs, we were unable to collect data on high-level ICPs as well as sedation other than propofol. It is difficult to see a simple resolution to this limitation, since optimal treatment of the patient in terms of ICP control and best practice sedation is naturally of the highest priority. Data collection is, however, ongoing, and respective cases may appear to further our understanding of how clinical factors act upon MEPs. Specifically, a standardized I/O curve for varying levels of propofol could be very useful in interpreting mapping results achieved under varying levels of sedation. Fifth and lastly, while the navigational aspect of nTMS enables reproducible targeting of specific cortical sites, the resulting MEPs are also influenced by aspects aside from cortical stimulation site. This could complicate longitudinal patient-specific monitoring of nTMS based parameters such as rMT. Examples for these factors include underlying EEG activity, prior activity of the target muscle or limb posture [48,49,50,51]. While these factors are known confounders of MEP replicability, other studies have shown that by sampling 20 or more trials, good inter-session reliability can be achieved regarding measures of motor cortex excitability [48,49]. This should be kept in mind for any clinical application involving longitudinal measurement of MCS excitability.

## 5. Conclusions

This study demonstrated the feasibility of nTMS motor mappings in the ICU setting. In so doing, we have shown how a variety of confounding factors inherent to the ICU setting may be mitigated. We have demonstrated that MEPs can be obtained in spite of propofol and opioid sedation, that electrical interference due to ICU equipment can be reduced to the point where mapping is possible, and that CT may be used successfully for nTMS navigation. Moreover, we have demonstrated how nTMS can be successfully integrated into routine workflow and intensive patient care. Our data further suggest that single-pulse motor mapping is safe in a range of different diagnoses commonly encountered in the neurological ICU. We have also presented three case studies in which nTMS mapping provided valuable insight into the neurological state of the patient, and two cases changed the clinical management. These results demonstrate a need for future studies to evaluate systematically the clinical benefits provided by the use of nTMS in patients with acute neurological injury.

## Figures and Tables

**Table 1:** Translation of navigated transcranial magnetic stimulation from outpatient setting to critical care setting.

Factor	Outpatient Setting	Critical Care Setting	Challenge
Imaging	High-resolution MRI as standard imaging	CT as most common imaging modality	Does CT offer comparable navigation to MRI?
Safety	Very rare occurrences of serious adverse effects (i.e., seizures)	No published data	Does nTMS safety extend to critical neurological damage?
Setup	Patient usually without monitoring, uninterrupted workflow	Monitoring and adjustment of vital parameters required	Is nTMS compatible with multimodal monitoring and clinical workflow?
EMG	Little environmental background noise	High amount of background noise	Can EMG noise be adequately reduced for nTMS motor mapping?
Patient	Patient is usually awake, cooperative, with little impairment	Sedation, hemicraniectomy, ICP elevation	What is the influence of clinical factors on MEP evokability?

This table gives an overview over challenges in translating navigated transcranial magnetic stimulation (nTMS) motor mappings from the outpatient to the critical care setting. Other abbreviations: magnetic resonance imaging (MRI), computed tomography (CT), electromyography (EMG), motor evoked potential (MEP), intracranial pressure (ICP).

**Table 2:** Patient overview.

Patient	Age	Sex	Principal Diagnoses	Etiology	MEPs Elicited
01	69	m	Osteomyelitis of cervical spine	Chronic	Yes
02	45	m	SAH, SDH	Fall	Yes
03	76	m	SDH	Fall	Yes
04	82	m	Central Cord Syndrome	Fall	Yes
05	74	f	SDH	Fall	Yes
06	89	f	Central Cord Syndrome	Fall	Yes
07	64	m	Occlusion of ICA	Spontaneous	No
08	61	m	SAH, SDH	Fall	Yes
09	17	f	TBI	Vehicle accident	Yes
10	66	m	Central Cord Syndrome	Assault	Yes
11	49	m	SDH, IPH	Unknown	No
12	57	m	SDH	Fall	Yes
13	77	m	C2 fracture	Fall	Yes
14	75	f	Cervical spine injury	Vehicle accident	Yes
15	79	m	Spinal metastases	HCC	Yes
16	71	f	Spinal cord injury C1-C7	Fall	Yes
17	77	m	SDH	Fall	Yes
18	39	m	SDH	Assault	Yes
19	70	f	Hemorrhagic Stroke	Spontaneous	No
20	40	m	SDH	Unknown	Yes
21	67	m	C1 fracture	Assault	Yes

This table shows an overview over all patients included in this study. Abbreviations: subarachnoidal hemorrhage (SAH), subdural hemorrhage (SDH), internal carotid artery (ICA), traumatic brain injury (TBI), intraparenchymal hemorrhage (IPH).

## Supplementary Materials

The following are available online at https://www.mdpi.com/2076-3425/10/12/1005/s1, Document S1: Using cranial CT to construct a headmodel in Nexstim NBS.

## Abbreviations

CT	Computed tomography
DCS	Direct cortical stimulation
ECG	Electrocardiography
EEG	Electroencephalography
EMG	Electromyography
fMRI	functional magnetic resonance imaging
ICA	Internal carotid artery
ICP	Intracranial pressure
ICU	Intensive care unit
IPH	Intraparenchymal hemorrhage
MCS	Motor cortical system
MEG	Magnetoencephalography
MEP	Motor evoked potential
MRI	Magnetic resonance imaging
nTMS	Navigated transcranial magnetic stimulation
rMT	Resting motor threshold
SAH	Subarachnoidal hemorrhage
SDH	Subdural hemorrhage
TBI	Traumatic brain injury
TMS	Transcranial magnetic stimulation
