# Supplementary material for: Navigated TMS in the ICU: Introducing Motor Mapping to the Critical Care Setting
Source: Brain Sci. 2020 Dec 18;10(12):1005. doi: 10.3390/brainsci10121005 (PMC7765929; doi:10.3390/brainsci10121005)
Supplement: Supplementary file 1 [file brainsci-10-01005-s001.pdf]

nTMS in the ICU: Introducing Motor Mapping to the Critical Care Setting

Supplementary Document S1

Using cranial CT to construct a headmodel in Nexstim NBS

This workflow details a confirmed method for system 4.0 and 5.0. Other parameters than the ones given may work equally.

1. Acquiring the data:

The system accepts CT series with slice thickness from 2 mm to 0.6 mm, as long as total data size remains below roughly 14.5 MB.

Export the study as a series of DICOM files. Before proceeding to the next steps, the slices should appear analogous to the following example:

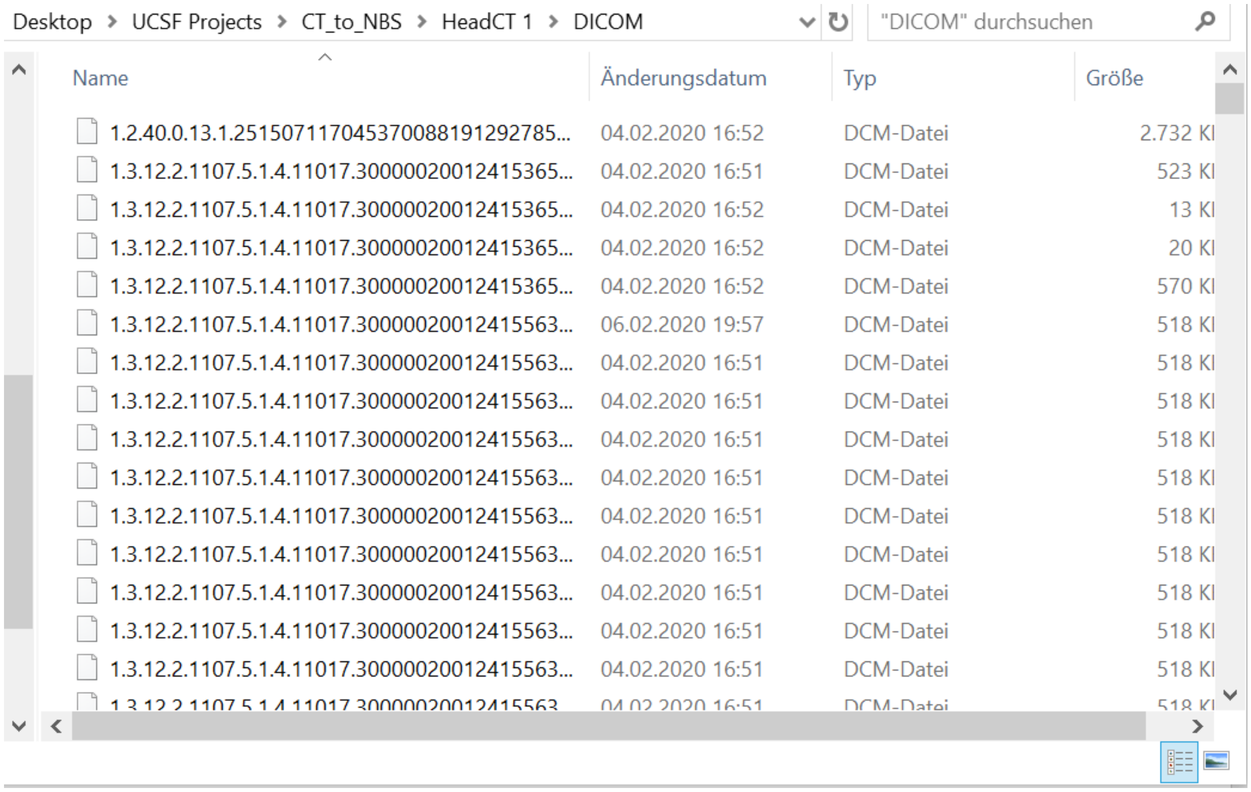

Fig. 1: Example of CT exported in DICOM format.

2. Editing the CT:

A CT series may contain elements that hinder the reconstruction of an optimal head model, such as e.g. a headrest, digital objects such as an orientation cube and others.

To ensure a usable CT, these elements must be removed via editing. For this, the freeware Aliza was used [available under https://www.aliza-dicom-viewer.com/home].

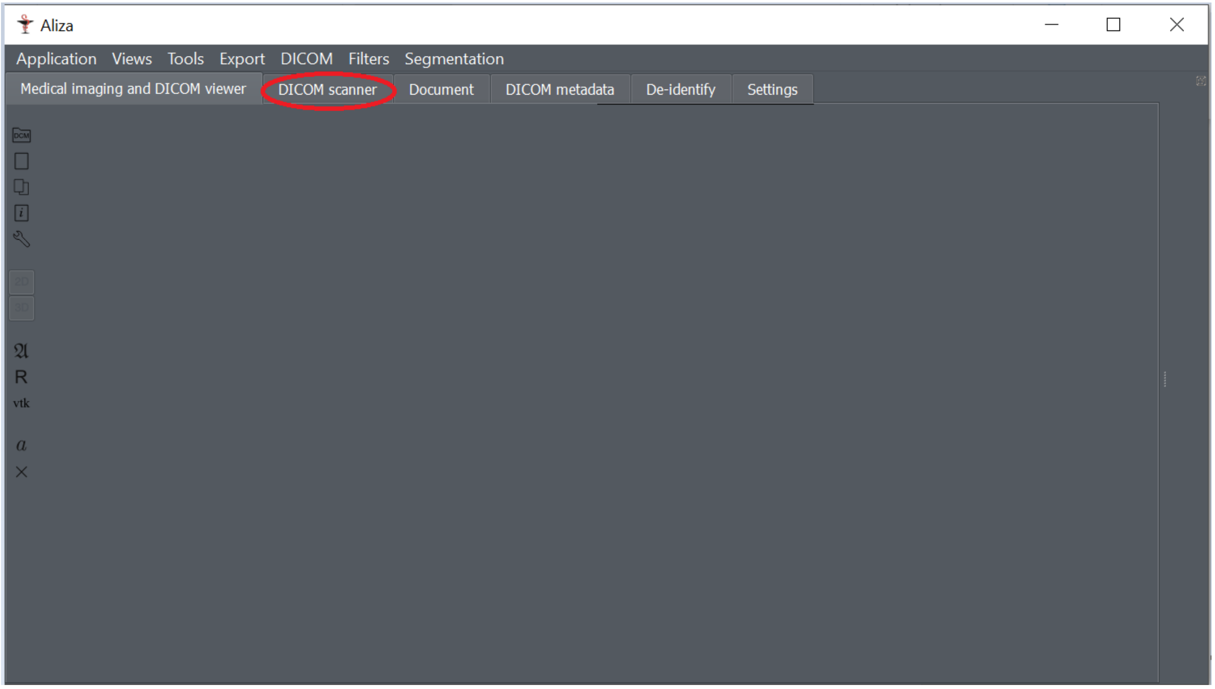

Fig. 2: Aliza GUI, with loading option marked out.

To load DICOM series, open the section “DICOM scanner” as pictured in Fig. 2. The option “Select directory” will lead to loading of the DICOM series. The result should look analogous to the following example:

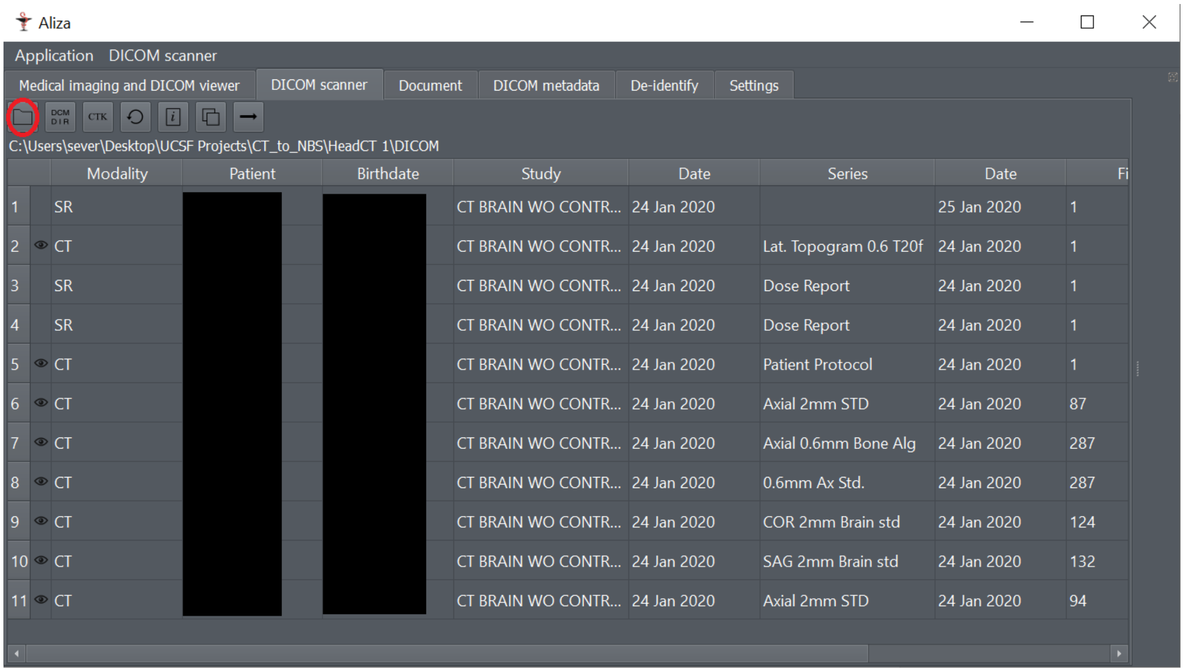

Fig. 3: Multiple imported CT scans. Option “Select directory” marked with red.

Select the desired CT study from the ones present. Double click to load.

After loading, both the CT slice view and a 3D reconstruction of the head should be visible (Fig. 4).

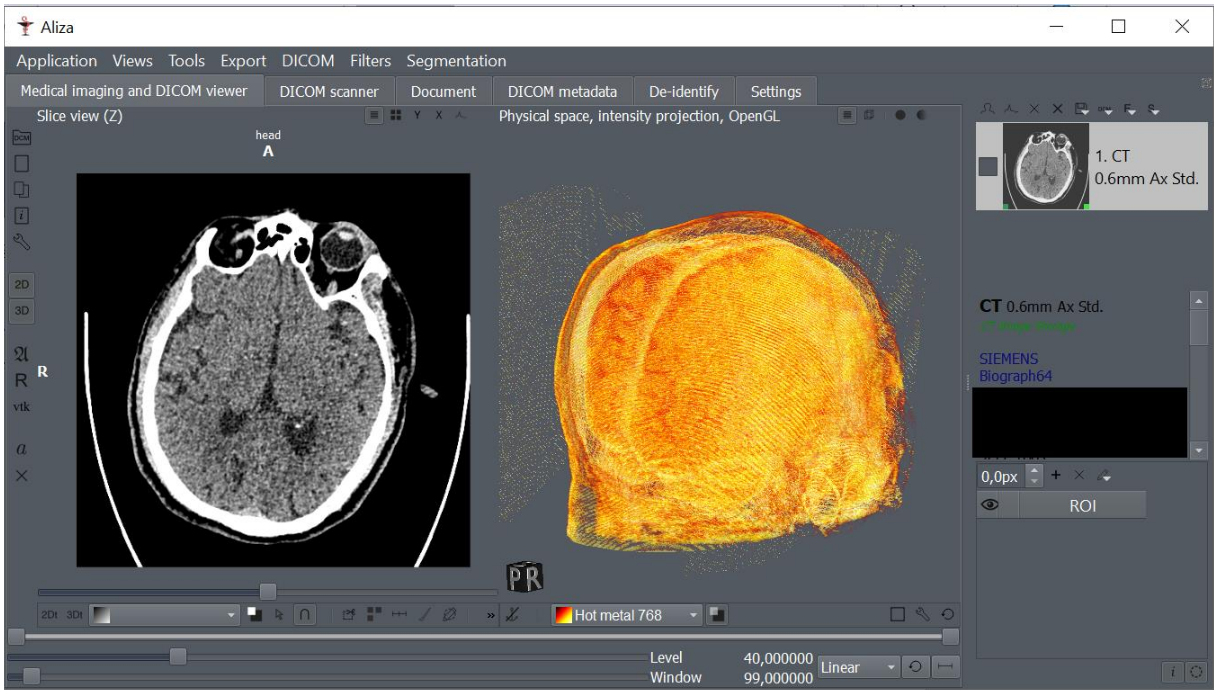

Fig. 4: Loaded CT, ready to be edited. Note the hyperdense headrest around the skull.

To remove subsections of the scans such as the headrest, “Tools” -> “Select sub-image” -> “X, Y – selection rectangle” may be used (Fig. 5).

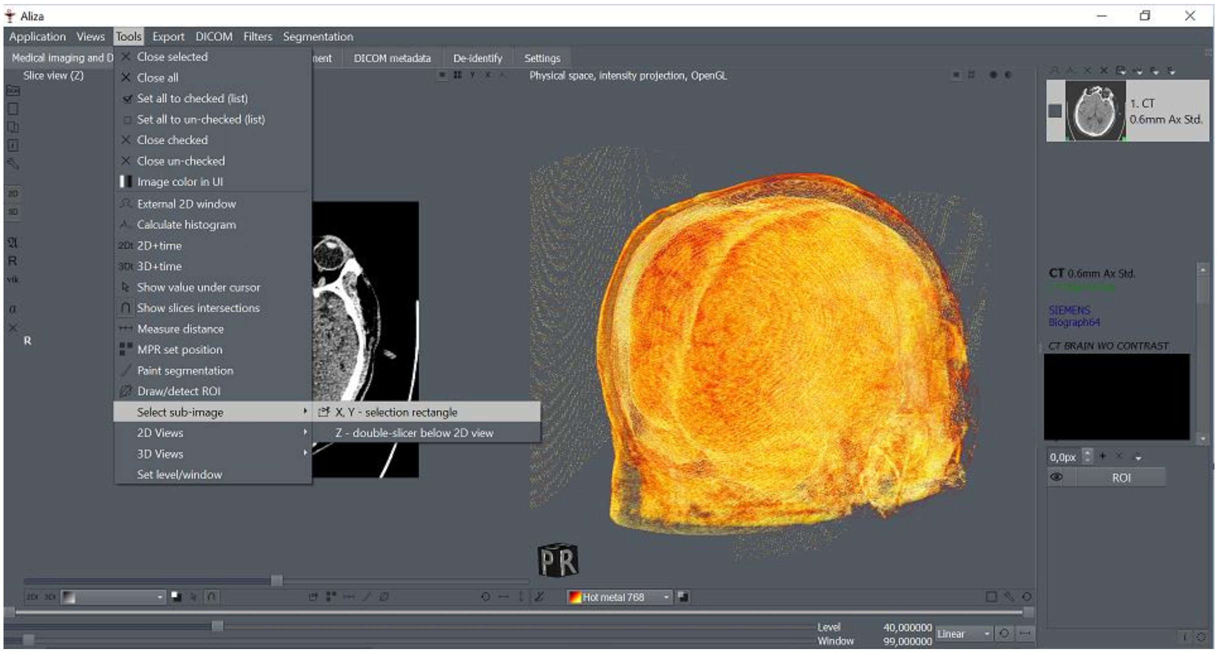

Fig. 5: Location of selection frame

The selection frame and the option “Filters” -> “Erase selected region” can be used to sequentially remove problematic elements from the series (Fig. 6). This should also erase any digital objects embedded into the series (e.g. orientation cube). Care should be taken not to erase any actual anatomy by mistake.

The 3D model on the right side may be helpful for checking whether every part of the headrest has been eliminated.

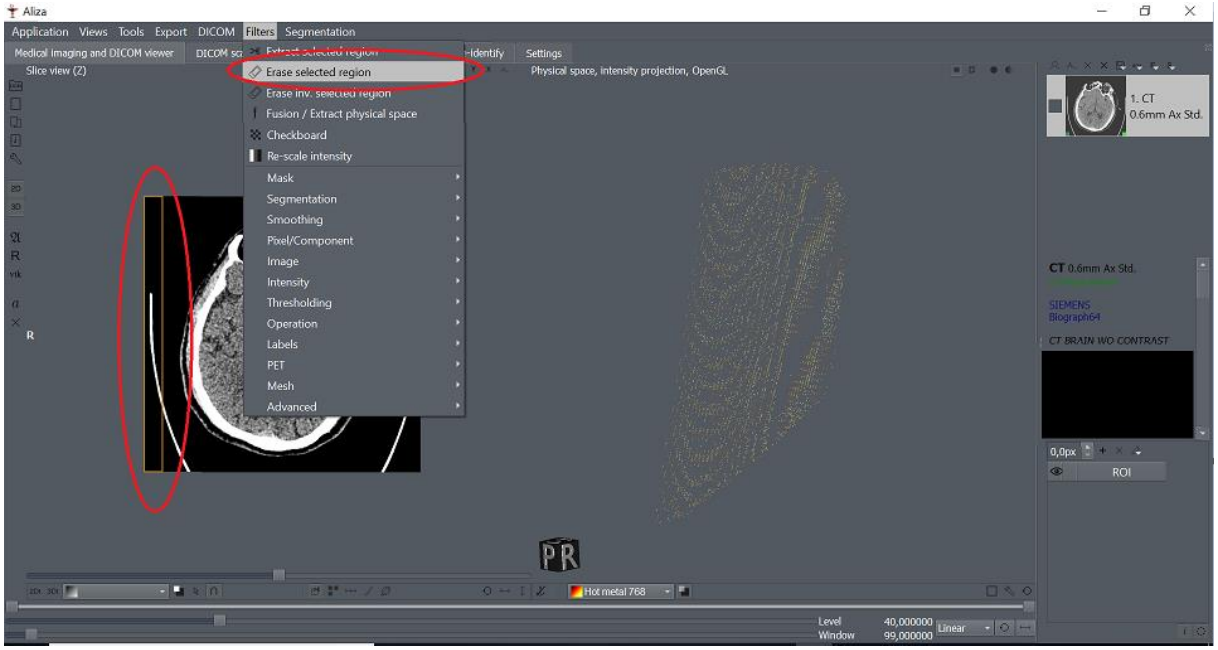

Fig. 6: Erasing a selected section out of the CT scan.

3. Rescaling the CT intensities

After complete removal of hindering objects, the CT must be windowed appropriately and rescaled.

For windowing, good results were observed with placing the level at 38 and window width at 584. This is needed to ensure good visibility of the skin for the head model.

If windowing is done without enough emphasis on scalp visibility, the NBS system will reconstruct a model unsuitable for registration.

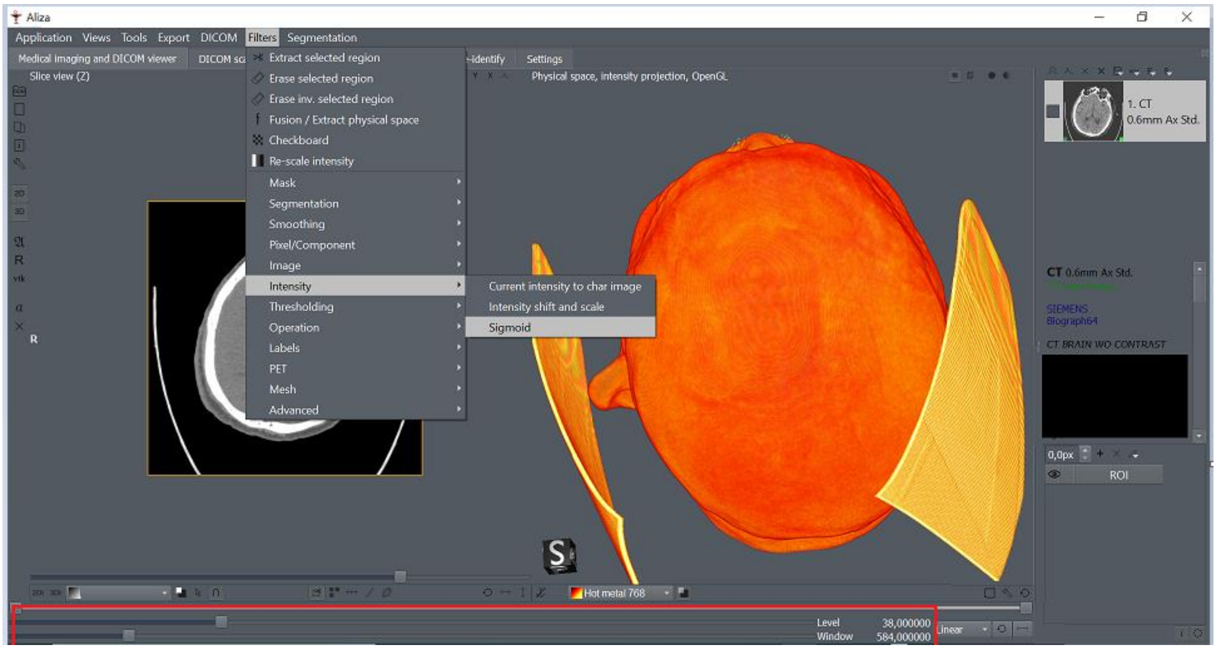

Fig. 7: Ideal windowing and location of Sigmoid intensity scaling option. Note that in this showcase, the headrest is not removed. This should ideally be done before rescaling.

For rescaling, the Sigmoid option has proven viable ("Filters" -> "Intensity" -> "Sigmoid"; Fig. 7).

For Sigmoid rescaling parameters, set Output minimum to 0 and Output maximum to 255.

4. Exporting the CT

Export should be performed with "Export DICOM CT, MR series" (Fig. 8).

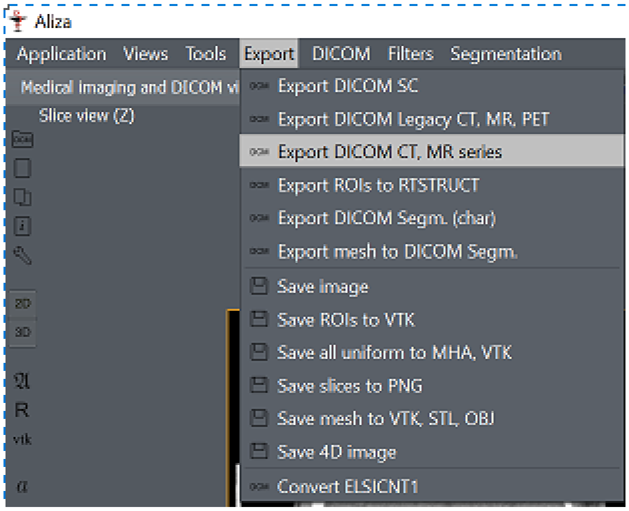

Fig. 8: Location of export option.

Importantly, to ensure that the export is viable, select the option “Lossless JPEG2000” for preferred Transfer Syntax (Fig.9).

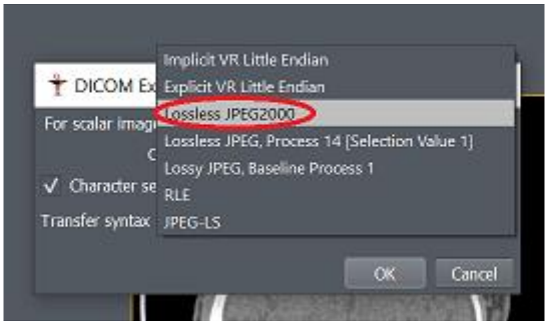

Fig. 9: Preferred Transfer Syntax

The resulting export should yield a functioning head model when used as input for the NBS system.
